# Technical Intraoperative Maneuvers for the Management of Inferior Vena Cava Thrombus in Renal Cell Carcinoma

**DOI:** 10.3389/fsurg.2017.00048

**Published:** 2017-09-06

**Authors:** Dionysios Dellaportas, Nikolaos Arkadopoulos, Ioannis Tzanoglou, Evgenios Bairamidis, George Gemenetzis, Pantelis Xanthakos, Constantinos Nastos, Georgia Kostopanagiotou, Ioannis Vassiliou, Vassilios Smyrniotis

**Affiliations:** ^1^2nd Department of Surgery, Aretaieion University Hospital, University of Athens School of Medicine, Athens, Greece; ^2^4th Department of Surgery, Attikon University Hospital, University of Athens School of Medicine, Athens, Greece; ^3^2nd Department of Anesthesiology, Attikon Hospital, University of Athens School of Medicine, Athens, Greece

**Keywords:** hepatic veins, inferior vena cava, neoplastic thrombi, renal cell carcinoma, renal vein

## Abstract

**Introduction:**

Renal vein or inferior vena cava (IVC) invasion by neoplastic thrombus in patients with renal cell carcinoma (RCC) is not an obstacle for radical oncological treatment. The aim of this study is to present our technical maneuvers for complete removal of the intracaval thrombus without compromising hemodymanic stability of the patient.

**Materials and methods:**

Between 2000 and 2014, 15 RCC patients with IVC involvement of levels I–III were treated with curative intent and were prospectively studied. The operative technique varied according to thrombus extent. For type I, extraction of the thrombus is facilitated by a 2–3 cm longitudinal incision on the IVC that begins at the level of the renal vein and extends cranially, encompassing a vessel wall rim of the orifice of the resected renal vein. For type II cases, the IVC is clamped above the neoplastic thrombus, and for type III, the IVC clamping is combined with hepatic blood flow control with “Pringle maneuver.” For type IV, the IVC is clamped above the diaphragm, or if the thrombus extends into the right atrium cardiothoracic input is appropriate.

**Results:**

The main operative steps include preparation and control of the renal vessels and the IVC. Occasionally, for type III tumor thrombi, the patient becomes hemodynamically unstable when IVC is clamped suprahepatically. In such a case, a novel operative maneuver of milking the thrombus below the orifice of the hepatic veins, and subsequently the IVC clamp also beneath the hepatic veins, allowing release of the “Pringle maneuver” is performed. This operative step restores hepatic blood flow and hemodynamic stability and is based on the floating nature of the thrombus into the IVC. Mean operative time was 120 min (range from 90 to 180 min), and average liver and renal warm ischemia time was 20 min (range from 15 to 35 min). Postoperative overall hospital stay ranged from 7 to 13 days.

**Conclusion:**

The technical solutions employed in the current study allow successful removal of neoplastic thrombi from the IVC in most cases, associated with minimal perioperative complication rate even for patients who due to multiple comorbidities would be considered otherwise inoperable.

## Introduction

About 4–10% of renal cell carcinoma (RCC) patients present with invasion of the renal vein or the inferior vena cava (IVC) with neoplastic cells, forming a thrombus (1). Surgical management of these tumors is challenging and associated with significant morbidity and mortality (2), while neoplastic pulmonary embolus is reported in 2–3.4% of cases (3). RCC thrombus in the large veins is a floating neoplastic lesion. Invasion or dense attachment to the wall of the vessels are rare incidents, and radical nephrectomy combined with extraction of the neoplastic thrombus is an oncologically sound approach that can result in long-term survival, even in cases with distant metastasis (4, 5). The most crucial technical step of the procedure is IVC control and prevention of tumor thrombus fragmentation. A classification system of the IVC thrombus is commonly used for RCC patients, according to the level of thrombus extension in relation to the orifices of the hepatic veins (6). At level 0, the thrombus extends to the renal vein only; at level I, the neoplastic emboli extends into the IVC to no more than 2 cm above the renal vein; at level II, the thrombus reaches into the IVC to more than 2 cm above the renal vein but not to the hepatic vein; moreover at level III, the thrombus reaches into the IVC above the hepatic veins but not above the diaphragm; and finally at level IV, the thrombus extends into the supradiaphragmatic IVC or the right atrium.

The aim of this study is to analyze our technical maneuvers for complete removal of the intracaval thrombus without compromising hemodymanic stability of the patient even in cases with intrathoracic IVC tumor embolus extension into the intrathoracic IVC.

## Materials and Methods

**Figure 1:**
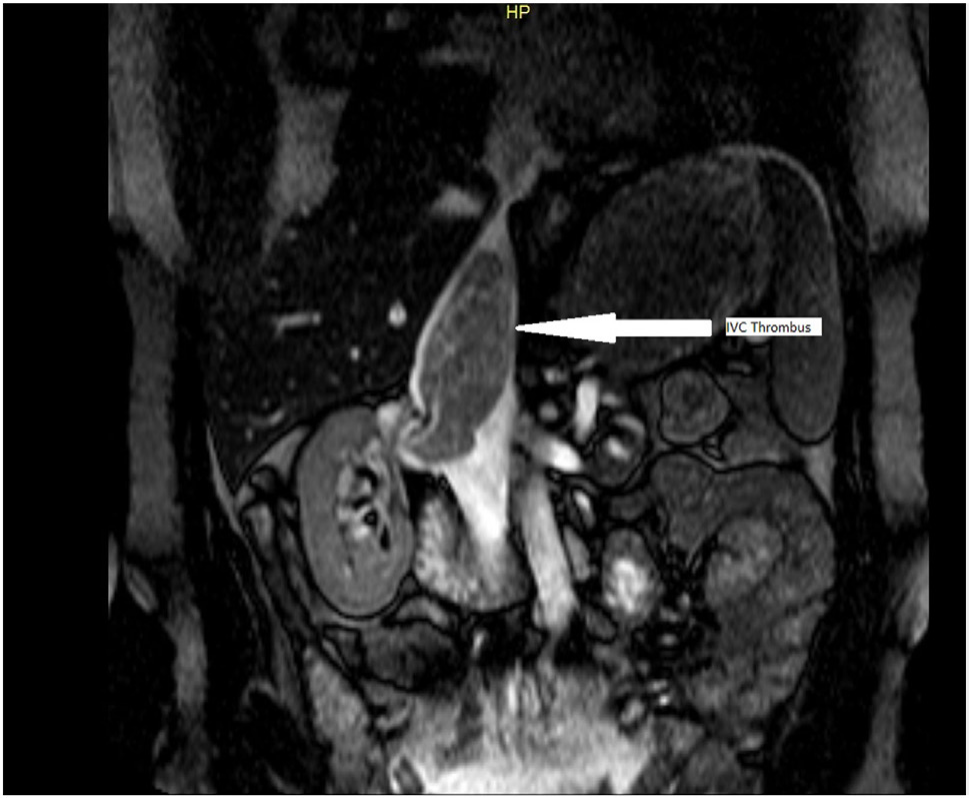
Magnetic resonance imaging image showing extension of the thrombus from the right kidney into the inferior vena cava (IVC) up to the level of the diaphragm, arrow showing IVC thrombus.

From January 2000 until December 2014, 15 patients with clear cell RCC and IVC involvement were treated in a single tertiary center. Laparotomy was the preferred approach for all case with no need of cardiopulmonary bypass (CPB). All operations were carried out by the same surgical team, and VS was the senior operating surgeon. There were 10 male and 5 female patients, median age 61 years (range 39–72 years of age). Type I involvement of the IVC was documented in six patients, type II in five patients, and type III in four cases. Abdominal and chest ultrasonography (U/S), computed tomography (CT) of chest and abdomen as well as CT angiography, were used preoperatively for staging and identification of caval involvement level. Magnetic resonance imaging (MRI) was also used in equivocal cases, because it is currently the gold standard for detecting the thrombus level (Figure 1). The reported sensitivity of MRI approaches 100% regarding this part of the staging process (7).

Operative time, estimated blood loss, number of packed red blood cells (pRBCs) transfusions, as well as postoperative complications, hospital stay, re-admissions, histopathological findings, and survival parameters were recorded for all patients. No patient had metastatic disease, and all operative procedures had curative intent.

All procedures started with the patient in the supine position and either a right subcostal incision with midline extension (J shape) for right-sided tumors or bilateral subcostal (rooftop) with midline incision (Mercedes) for left-sided neoplasms. After retractor placement and initial evaluation for the presence of intraabdominal metastatic disease, the surgeon’s first concern is the isolation of the infrarenal IVC. The latter is prepared cautiously, and a vessel loop is placed 1–2 cm below the confluence of the renal veins with the IVC where no retroperitoneal or lumbar vein branches are present. For right-sided tumors, the left renal vein is encircled with a vessel loop to secure its control during tumor thrombus extraction, while the left renal artery may be left unclamped, since the left kidney possess rich collateral vein circulation (8). On the other hand, for left-sided tumors, the surgeon must control both the inflow and outflow of the right kidney to avoid significant hemorrhage during IVC opening.

According to the classic technique for type I neoplastic IVC thrombus, confirmation of the extension of the embolus is done using intraoperative U/S along with manual palpation and afterward a fine vascular clamp is placed above the intraluminal IVC growth. The vessels of the healthy kidney and the infrarenal IVC are also temporarily clamped. Thrombus entrapment is achieved, and a radical nephrectomy is carried out, en bloc with the thrombus. Extraction of the thrombus is facilitated by a 2–3 cm longitudinal incision on the IVC that begins at the level of the renal vein and extends cranially, encompassing a vessel wall rim of the orifice of the resected renal vein. After specimen extraction, the IVC is repaired in a bloodless surgical field with a continuous polypropylene 4-0 suture. Deairing the vena cava is performed, unclamping the infrarenal IVC, using needle aspiration through the cavotomy and before securing the IVC repair final suture knot. Removal of the clamps from the suprarenal IVC and the vessels of the healthy kidney and meticulous hemostasis is the final step of the operation. Trendelenburg positioning of the patient was used when central venous pressure was almost 0; however, otherwise this maneuver was not essential.

**Figure 2:**
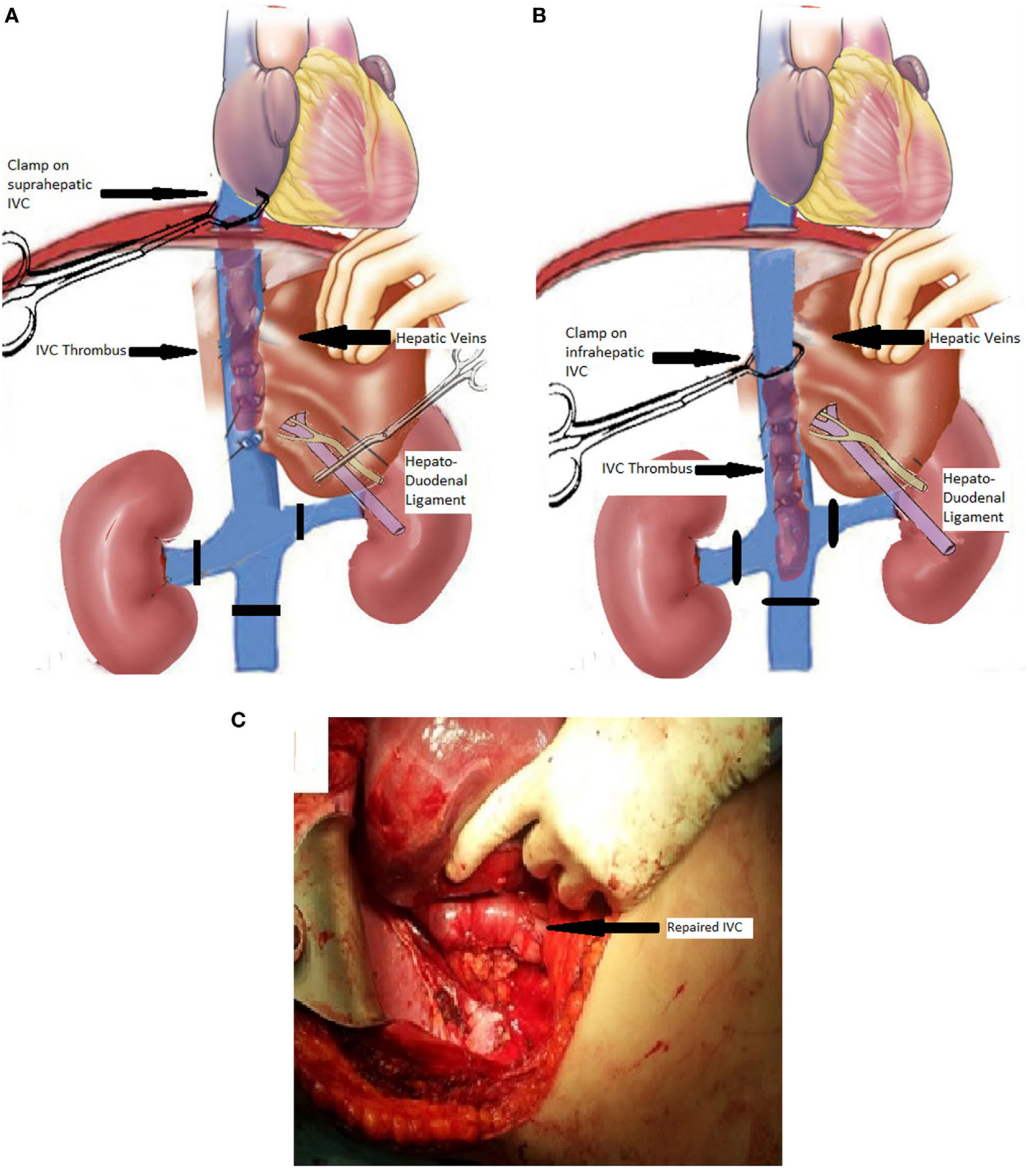
Schematic view of the procedure in a patient with type II involvement of the inferior vena cava (IVC) with thrombus extending from a right-kidney carcinoma. **(A)** Schematic view of the IVC, renal veins, liver, hepatic veins, and hepatoduodenal ligament (portal triad structures). The infrarenal IVC and the left renal vein have been encircled with vessel loops (black lines) to allow for subsequent vascular control. A vascular clamp has been placed on the suprahepatic IVC, and the hepatoduodenal ligament has been clamped. **(B)** The suprahepatic IVC clamp has been exchanged with an infrahepatic IVC clamp after milking the thrombus downwards, and the hepatoduodenal ligament clamping (Pringle maneuver) has been released for hemodynamic stability. **(C)** Full view of the repaired retrohepatic IVC at the end of the procedure (arrow showing the repaired IVC).

In type II and III patients, the operation starts again with control of the infrarenal IVC and the renal vessels as described earlier; however, the cranial extension of the thrombus at the level or even beyond the orifices of the hepatic veins defines our approach to IVC control. Briefly, without touching the IVC, the coronary ligament is rapidly divided, and the confluence of the hepatic veins with the IVC is reached and visualized. The exact location of the thrombus is assessed with U/S and careful manual palpation, and an important decision is made: if no tumor is present above the hepatocaval junction then a proper Satinsky clamp is placed on the suprahepatic IVC incorporating a small part (1–2 cm) of diaphragmatic muscle (Figure 2A). The hepatoduodenal ligament is already occluded (Pringle maneuver), right before the above step (Figure 2A). Rapid division of the right liver lobe ligaments follows and rotation of the right liver, toward midline provides excellent view of the retrohepatic IVC. Continuous communication between the operative team and the anesthesiologist is of paramount importance, and evaluation of the hemodynamic stability is crucial in intraoperative decision making. If the patient does not become unacceptably hypotensive then the procedure is fulfilled with the radical nephrectomy and tumor thrombus extraction as described for type I tumors. This was achieved in our series in four out of five type II cases. In the fifth case, hemodynamic instability forced us to come up with a modified plan. With the retrohepatic IVC prepared, the surgeon tried to manually using two fingers and milking movements displace the apex of the floating tumor thrombus caudally, below the orifices of the major hepatic veins. The suprahepatic IVC clamp was exchanged with another, right underneath the confluence of the right hepatic vein and above the neoplastic thrombus, while the portal triad occlusion was released (Figure 2B). By this creative surgical maneuver, 25% of the IVC blood flow, which is coming from the liver, was maintained and hemodynamic parameters improved, allowing safe continuation of the operation with a good final result (Figure 2C).

For type III patients, where the neoplastic thrombus extension is cranially up to the level of the intrathoracic IVC, just below the right atrium, the operation is once again initiated with infrarenal IVC and contralateral renal vessels control as described earlier. As in type II cases, the suprahepatic hepatocaval junction is exposed and meticulously prepared. If the Satinsky diaphragmatic clamp is placed blindly, without securing that the apex of the thrombus is below the clamp, then fragmentation and neoplastic pulmonary embolism may occur. That is why intrathoracic control of the IVC through the abdomen is attempted. The diaphragm is opened up at its tendonous central portion, with a horizontal 2–3 cm incision, above the hepatocaval junction, providing exposure of the intrathoracic IVC and the right atrium. The position of the apex of the thrombus is determined by U/S and finger palpation, and once again the surgeon takes advantage of the floating nature of the thrombus, by milking it downwards and applying a fine clamp on the IVC at the diaphragmatic level. It has to be mentioned that in this case scenario, ligation of the phrenic veins bilaterally is needed, because if not, when the IVC is opened for tumor thrombi extraction, troublesome hemorrhage occurs. In three type III cases, where hemodynamic stability was maintained, the whole procedure continued and was completed with no further concerns; however, the right lobe of the liver was also mobilized, and the retrohepatic IVC was visualized, just in case the variation described earlier for type II patients was needed. Indeed, in the final type III case, mild hypotension occurred, and due to anesthetic concerns, because of patient’s comorbidities, the surgeon managed to milk the neoplastic thrombus further caudally, below the orifices of the hepatic veins. The latter maneuver allowed exchange of the “diaphragmatic” clamp of the IVC, with one just below the hepatic veins, and release of “Pringle maneuver,” which led to restoration of hemodynamic stability. We tend to leave the diaphragmatic incision open and create a small pericardial window, for drainage of pericardial collections, in cases of intrathoracic IVC handling.

## Results

All 15 patients underwent a successful radical nephrectomy and extraction of the neoplastic IVC thrombus (Table 1). In one patient, the procedure was combined with left colectomy, while in another patient distal pancreatectomy and splenectomy were also needed for oncological reasons. Tumor-free resection margins were achieved in all cases. Mean operative time was 120 min (range from 90 to 180 min), and average liver and renal warm ischemia time was 20 min (range from 15 to 35 min). Intraoperatively only three of the patients were transfused with 2–6 U of pRBCs. Postoperative overall hospital stay ranged from 7 to 13 days, with a median of 9 days. No major complications occurred postoperatively, apart from two respiratory tract infections and a pleural effusion in the patient with the concomitant distal pancreaticosplenectomy. One patient with type II IVC thrombus, which had been diagnosed preoperatively with distal pulmonary embolism, developed respiratory failure and died in the immediate postoperative period. Autopsy was performed and confirmed the presence of second fresh pulmonary emboli, which histopathological examination revealed that it was neoplastic. It has to be assumed that this process was a result of the intraoperative maneuvers. There were no re-admissions in any of our patients.

**Table 1:** Demographic and operative data.

Patient no.	Sex/age	Inferior vena cava thrombus classification level	Histopathological staging (AJCC, TNM 7th edition)	Operative time (min)	Blood loss (mL)	Postop. complications	ICU stay (days)	Hospital stay (days)
1	M/55	I	pT3bN0M0	100	200	n/a	1	7
2	M/61	I	pT3bN1M0	90	300	n/a	1	8
3	F/57	I	pT3bN1M0	95	200	Respiratory tract infection	2	7
4	M/52	I	pT3bN0M0	90	200	n/a	1	7
5	M/67	I	pT3bN0M0	90	200	n/a	1	10
6	F/68	I	pT3bN0M0	90	300	n/a	1	7
7	F/65	II	pT3bN1M0	120	800	n/a	2	9
8	M/39	II	pT4N1M0	180	1,500	Pleural effusion	4	13
9	F/55	II	pT4N0M0	110	500	n/a	2	8
10	F/69	II	pT3bN0M0	115	300	n/a	2	7
11	M/71	II	pT3bN1M0	140	300	n/a	1	7
12	M/72	III	pT3cN1M0	170	900	Respiratory tract infection	2	10
13	M/64	III	pT4N0M0	150	400	n/a	1	8
14	M/59	III	pT3cN1M0	120	900	n/a	1	9
15	M/61	III	pT3cN1M0	140	300	n/a	2	Postop. death

*M, male; F, female; n/a: not applicable*.

Follow-up ranged from 12 up to 72 months and late deaths occurred from regional (one patient) or distant metastatic recurrence (nine patients), while for four patients there were no long-term follow-up data. One-year survival was 93%.

## Discussion

Natural history of RCC includes the formation of a neoplastic thrombus in about 4–10% of cases. The thrombus propagates into the ipsilateral renal vein or even the IVC (9). Even for this group of patients, 5-year survival rates after radical nephrectomy and complete tumor thrombus extraction has been reported to be 32–64% (4, 6, 10). Although, the proximal extent of the neoplastic thrombus may have a role as a prognostic factor (11), thrombus extraction in conjunction with radical nephrectomy is considered the standard of care. Tyrosine kinase inhibitors, sunitinib, and sorafenibare also being used in the adjuvant and neoadjuvant setting for RCC patients, providing longer survival rates, disease free intervals, and subsequently expanding the indications for aggressive surgical efforts (12).

In the literature surgical approach for level III and IV cases usually is combined with CPB and hypothermic circulatory arrest, a procedure that requires sternotomy and may be complicated with platelet dysfunction, coagulopathy, multisystem organ failure and generally increased morbidity in comparison with abdominal only approaches (13). Despite these risks, in type IV cases or in rare patients with strong adherence of tumor thrombus to the IVC wall, CPB is required. Our experience emphasizes that level I–III patients can be approached entirely transabdominally, and using the right liver mobilization as routine part of the operation facilitates an excellent control of the retrohepatic IVC. Especially, in cases when the IVC is clamped and intraoperative hypotension precludes the safe continuation of the procedure, careful finger milking of the thrombus apex downwards, below the major hepatic veins orifices is achievable. This maneuver simplifies the operation, as long as the superior clamp of the IVC is moved below the major hepatic veins and occlusion of the liver inflow with “Pringle” maneuver is no longer needed. The whole maneuver is based on the fact that anatomically there is about 1 cm of IVC length below the orifice of the major hepatic veins that has no venous branches and thus there is no fear of injuries and major hemorrhage during the clamp application on the IVC. Of course, intraoperative U/S is of paramount importance for the identification of the thrombus extension into the IVC. Intraoperative U/S is also used for confirmation of the vascular anatomy along the retrohepatic IVC to avoid tearing of venous branches and significant blood loss. It has to be emphasized that some of these neoplastic emboli can be adherent to the IVC endothelium, requiring an “endarterectomy” type resection; however, in this series such a resection was never required.

We strongly suggest that this surgical technique described earlier reduces the perioperative complication rate, the intensive therapy unit stay, and in extension the overall hospital stay, making the operation appropriate even for patients with multiple comorbidities, considered otherwise inoperable.

In previous reports, a similar approach has been described (14, 15), with emphasis to the fact that liver transplantation techniques are commonly applied in IVC tumor thrombus manipulation, and “piggyback” mobilization of the liver is not always required for safe control of the neoplastic thrombus apex into the IVC. Our point is that the whole procedure can be performed rapidly, only with right liver lobe mobilization toward the midline and decision making should be prompt, while surgical and anesthetic team communication is “sine qua non” for the successful result. The surgical steps for the radical nephrectomy may be performed in the beginning of the surgical exploration, with “non-touch” surgical philosophy of the tumor site, and before the application of IVC control. This will allow quick removal of the specimen after IVC occlusion to prevent hemodynamic changes. In other reports, preoperative placement of an IVC filter is advocated as a measure to prevent a lethal embolic event resulting from intraoperative thrombus fragmentation (16). We feel that this measure is unnecessary and may endanger the incorporation of the filter into the thrombus. Also, the use of transesophageal echocardiography in cases of type III–IV tumor thrombus may give real time information for the proper manipulation of the neoplastic emboli.

In conclusion, RCC tumor thrombus into the IVC should be managed aggressively, and the transabdominal approach is adequate for complete tumor resection. Even for level III or high level II, neoplastic IVC thrombi retrohepatic IVC exposure and careful displacement of the thrombus downwards may allow IVC occlusion below the major hepatic veins orifices, and better hemodynamic control during a complex procedure.

## Ethics Statement

The current study has been approved from Aretaieion University Hospital and Attikon University Hospital Ethics Committee.

## Author Contributions

DD drafted the manuscript, analyzed the data, and revised the article; NA critically analyzed the data and designed the study; IT and EB constructed the database and helped with manuscript drafting; GG and PX analyzed the data and reviewed current literature on the subject; CN edited all figures and helped with drafting and revising the manuscript; GK designed this study and guided all the authors in all stages of this paper; IV and VS were the senior surgeons performed the operations and had the original study idea, reviewed and finalized manuscript’s version. All the authors approved final version of this manuscript.

## Conflict of Interest Statement

The authors declare that the research was conducted in the absence of any commercial or financial relationships that could be construed as a potential conflict of interest.
